# Atypical Presentation of Pemphigus Vulgaris: Nail Involvement in a 20-Year-Old Male

**DOI:** 10.7759/cureus.53609

**Published:** 2024-02-05

**Authors:** Apostolos Katsiaunis, Shari R Lipner

**Affiliations:** 1 Department of Dermatology, Tufts University School of Medicine, Boston, USA; 2 Department of Dermatology, Weill Cornell Medicine, New York, USA

**Keywords:** nail dystrophy, onychodystrophy, nail changes, nails, onychomadesis, pemphigus vulgaris

## Abstract

Pemphigus vulgaris (PV) mainly causes blistering of the skin and mucous membranes, with nail unit involvement being rare. Nail involvement may serve as an indicator of disease severity. We present a case of a 20-year-old male with PV who had both cutaneous and nail findings, with nail changes corresponding with disease severity. The patient with biopsy-confirmed PV, on prednisone and mycophenolate, presented to the emergency department with an acute flare of PV and severe mandibular pain and lymphadenopathy. At follow-up in our outpatient department, the physical examination was significant for onychomadesis and onycholysis of the fingernails. Prednisone and mycophenolate dosages were increased, and rituximab infusions were initiated. Bullae and mucosal lesions resolved on the follow-up, and nail changes improved. This case appends an unusual perspective to the limited literature on PV-associated nail changes, especially in younger patients. It advocates for meticulous history taking and physical examination and supports a correlation between nail symptoms and PV disease severity.

## Introduction

Pemphigus vulgaris (PV) is an autoimmune vesiculobullous disease that causes blistering of the skin and mucous membranes [1]. Nail involvement is uncommon and underreported [2]. We describe a case of PV in a 20-year-old male who presented with onychodystrophy, corresponding with the severity of his oral and cutaneous lesions and improving with PV treatment.

## Case presentation

A 20-year-old male with a diagnosis of PV presented with painful oral and cutaneous lesions. Two months before his initial visit to our dermatology clinic, the patient underwent a punch biopsy, with histopathology showing suprabasal clefting and a dermal eosinophil-rich infiltrate. Direct immunofluorescence testing showed intracellular deposits of C3 and IgG in the epidermis, consistent with a diagnosis of PV.

Prior to presenting to our clinic, the patient was initially managed with 20 mg of oral prednisone daily and 500 mg of mycophenolate twice daily. Despite initial improvement, after one month of treatment, he presented to the emergency department with mandibular pain and edema, submandibular lymphadenopathy, and bullae and erosions posterior, soft and hard palate, cheeks, and gums. He met the systemic inflammatory response syndrome criteria, indicating a heightened state of inflammation based on physiologic parameters, including heart rate, respiratory rate, leukocyte count, and body temperature. He was given a 10-day course of oral amoxicillin/clavulanate, and prednisone was increased to 40 mg.

**Figure 1:**
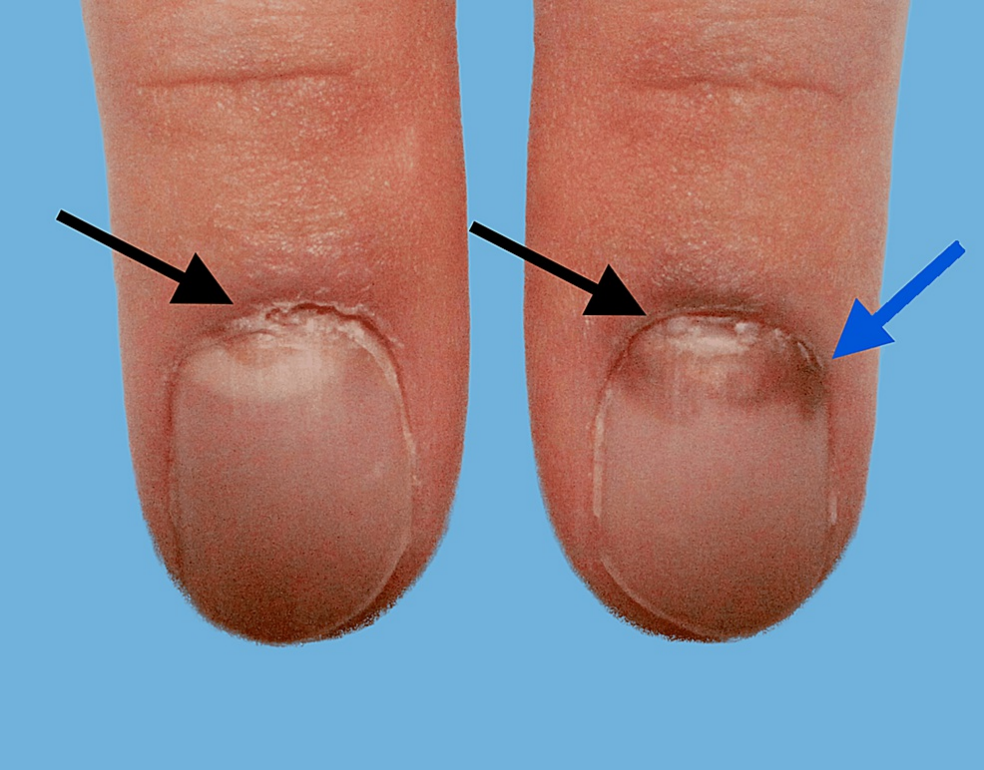
Clinical presentation of bilateral index fingernails at initial visit showing onychomadesis and brown discoloration of the nail plate proximally The black arrows indicate the presence of onychomadesis, while the blue arrow indicates brown discoloration of the nail plate.

On presentation to our dermatology clinic, three weeks after his prednisone dosage was increased, his bullae were healing, and he did not develop any new ones. His clinical examination was notable for hyperpigmented patches on his chest, back, abdomen, axillae, and upper medial thighs, corresponding to ruptured vesicles and bullae. No intact bullae were present. However, superficial ulcerations were observed in the bilateral buccal mucosa. There was onychomadesis, separation of the proximal and distal parts of the nail plate, and brown discoloration involving his fingernails (Figure 1), without involvement of the toenails.

**Figure 2:**
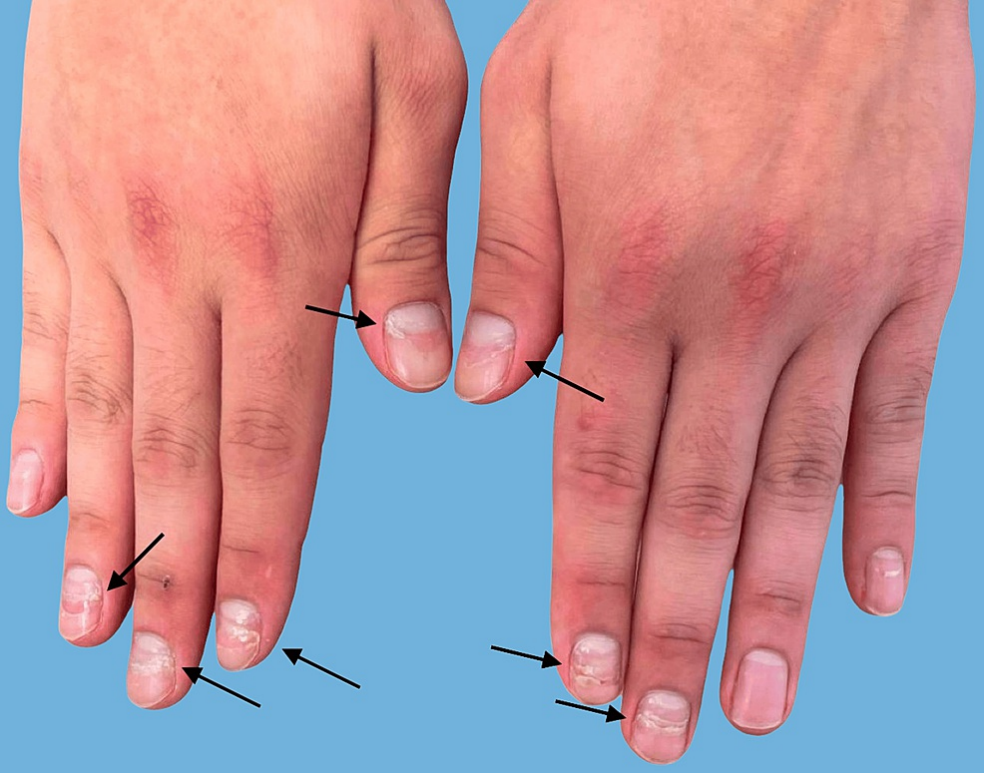
Clinical presentation of fingernails showing more distal onychomadesis The black arrows indicate the presence of onychomadesis.

The patient was started on sulfamethoxazole and trimethoprim 800 mg/160 mg three times per week, as well as omeprazole and a calcium vitamin D supplement. He was prescribed rituximab (1,000-mg IV infusion on Days 1 and 15), but the treatment was complicated by a grade 2 infusion reaction during the first infusion. He was continued on mycophenolate 500 mg twice daily and oral prednisone 40 mg daily, with a plan of decreasing his dose by 5 mg every two weeks. When he returned to the clinic for his two-week follow-up, he reported no new oral or cutaneous lesions and noted continued improvement in his existing lesions but not in his fingernail changes. At his seven-week follow-up, however, no developing onychomadesis was noted proximally, and the previously observed signs of onychomadesis had migrated distally, consistent with nail growth and clinical improvement (Figure 2). A comparison of clinical photographs at each visit helped evaluate nail improvement over time.

## Discussion

We report the case of a 20-year-old patient with PV who exhibited nail changes affecting his fingernails, corresponding to a flare in his PV. Nail changes associated with PV are either uncommon or underreported [2]. In a retrospective study conducted in the United States analyzing nail changes in patients with pemphigus (N = 141 patients), not limited to PV, 26% of patients had non-fungal nail changes that correlated with pemphigus disease severity, the most common being pachyonychia (25%), paronychia (25%), and onycholysis (21.5%) [2]. In PV patients, specifically, nail changes were present in 47% of cases. In a single-center retrospective observational study in Poland with 67 ethnically Polish patients, nail involvement was present in 13% of cases, with paronychia (13%), nail discoloration (10%), and Beau’s lines (7%) being the most common findings [3]. In a retrospective study with 79 patients in Iran, nail involvement was present in 32% of cases, with paronychia (10%) and onychomadesis (8%) being the most common changes [4]. In a case-control study in India involving 25 patients with PV, 80% exhibited nail changes, with paronychia (40%) and onychorrhexis (32%) being the most common findings [5]. In a case series of 15 patients with PV and nail involvement, paronychia was the most frequent, present in 60% of cases, followed by onychomadesis (33%) [6]. In a retrospective study conducted on 25 Egyptian patients with PV, the predominant nail changes were onycholysis and subungual hyperkeratosis, which were observed in 16 patients (64%) [7].

With PV-associated nail changes, fingernails are more often affected than toenails, particularly the first three, possibly because these are used more often and subject to local trauma [8]. In a case study of a 55-year-old female who experienced chronic, treatment-resistant paronychia, her onychodystrophy was the initial presentation of PV [9]. The onset of nail changes may precede a flare [10], present concurrently with mucosal and cutaneous lesions, or be the only symptom [7]. While nail changes often signify the progression of PV, they may also reflect treatment efficacy. For example, in a case report of a 34-year-old female, her PV-associated paronychia improved rapidly following treatment with oral tofacitinib and rituximab infusions [11]. In our patient, nail involvement presented with the initial PV lesions worsened with flare and improved with systemic treatment.

PV generally affects older individuals, most commonly in their fourth to sixth decade of life [12]. In a cross-sectional analysis of 1,795 adult pemphigus patients in the United States, not limited to PV, 78.5% of patients were at least 50 years old, and only 3.9% of adults with PV were under 30 years old [13]. Our patient is 20 years old, which makes his presentation of early-onset PV, in addition to his having associated nail involvement, atypical.

The cause of PV-associated nail changes and why they might be rare remains unclear, but it may be because the nail bed is an “immunologically privileged” site where the immune system is less active [9,14]. In the nail immune system, the quantity and function of antigen-presenting cells are markedly diminished compared to those in the cutaneous or mucosal epidermis. Furthermore, major histocompatibility class II and CD209 expression by Langerhans cells in the proximal nail matrix is notably decreased, along with decreased functionality and/or quantity of natural killer and mast cells around the human nail apparatus [8]. Additionally, it is hypothesized that the nail unit has a lower density, reduced expression, or relative sequestration of desmoglein 1 (DSG1) and DSG3 compared to the skin and mucosal epidermis. These are the proteins targeted by autoantibodies in PV. Furthermore, the nail matrix is devoid of a granular layer, and the nail bed epidermis is merely two to three cells thick [11]. As a result, the nail unit exhibits low levels of DSG1 and DSG3 expression. This low expression could serve as a protective mechanism, as a high concentration of autoantibodies targeting these desmosomal glycoproteins would be necessary for the nails to be affected in PV, potentially shielding the nail unit from autoimmune attacks [8,14].

While PV may present with nail changes, onychomadesis may stem from a wide range of etiologies. In the absence of characteristic PV symptoms or a known PV diagnosis, onychomadesis should prompt consideration of a broad differential diagnosis, including bacterial infections, viral illness, trauma, medication side effects, systemic disease, and idiopathic nail changes [15]. In this case, the patient’s established diagnosis of PV provided context for the observed nail changes. A limitation of our study is the possibility of conflating PV-related nail changes with similar presentations caused by other etiologies, emphasizing the importance of a thorough history taking and physical examination.

## Conclusions

This case of a 20-year-old patient with PV highlights the clinical significance of nail changes as an integral aspect of disease presentation and progression. Our findings underscore that nail involvement may be a clinical finding in patients with PV, although variably reported in the literature, and may correlate with disease severity. Recognizing nail changes as potential indicators of PV activity can lead to a more timely diagnosis and tailored management strategies. Our observations also contribute to the growing understanding of PV pathophysiology, particularly the role of the nail unit as an “immunologically privileged” site, potentially influencing the expression and impact of autoantibodies in PV.
